# On the Treatment of the Venereal Disease by Mercury

**Published:** 1838-01-01

**Authors:** 


### ON THE TREATMENT OF THE VENEREAL DISEASE BY MERCURY.

I. Practical Observations on the Venereal Disease and on the use of Mercury. By *Abraham Colles*, M. D., one of the Surgeons of Doctor Stevens's Hospital, and lately Professor of Surgery in the Royal College of Surgeons in Ireland. 8vo., Pp. 351. 1837.

II. The Works of John Hunter, F. R. S., with Notes. Edited by *James F. Palmer*, Senior Surgeon to the St. George's and St. James's Dispensary, &c., &c. In Four Volumes, Illustrated by a Volume of Plates, in Quarto. Vol. II. London, 1835.

We devoted some pages in the last number of this Journal to a highly important practical subject—the treatment of Syphilis by Mercury. If our readers will refer to the article in question,* they will find, or they may recollect that we examined the comparative merits of the mercurial and non-mercurial methods, and expressed our opinion in favour of the former. We then proceeded to two chapters of Dr. Colles's work—the one containing an account of the "Natural History of the Venereal Disease," the other occupied with a description of "Chancre" and of "Chancrous Sores."

We shall now resume the subject, in conformity with the pledge we gave, and we shall endeavour to present to our readers a series of notices on a disorder as frequent, as serious, and as difficult to be understood and treated, as any that afflict mortality.

It is much to be regretted, that Lock Hospitals are not more numerous and more extensive than they actually are in Great Britain. In the Metropolis of the Empire—a city with nearly two millions of inhabitants,—the solitary Lock Hospital is without funds to support patients, and without pupils to become acquainted with disease.

It is true that venereal wards exist in some of the General Hospitals. But they exist on a limited scale, and being subordinate to the other departments of the institution, they do not, and cannot, receive that degree of attention from the medical officers or from the students, which alone can qualify the former for enlarging our knowledge of the malady, or the latter for learning what is actually known.

The nature of venereal disorders, and the character of the generality of the patients, render their admission into General Hospitals a matter of questionable policy. The patients themselves can never be adequately separated from the common patients. Respectable females mingle with whores, and become familiar with vice. The moral pestilence, nay the physical disease, is constantly communicated, and the young girl who went into the hospital a simple maid, too often comes out an incipient prostitute. On the whole, it must be owned that the plan of appending venereal wards

* Pp. 379, et seq. No. 54.

to General Hospitals, has certainly done little good, and probably much evil. Science has not gained, and morality has suffered.

The reason why the Lock Hospital of London is unsupported may be readily divined. Conscientious people, the usual subscribers to charitable institutions, are averse to maintaining one which they conceive holds out impunity and a premium to vice. They think that syphilis, unlike many maladies, is a direct and proper punishment for sin, and that the hand of pity should not be extended to the sufferer from a guilty act.

We respect the motives, while we cannot concur with the reasoning of such persons. We must take the world as it is, not as it should be. If allowing persons of the lowest class to shift for themselves when affected with venereal maladies, would in any degree discourage vice and prevent such maladies, the refusal to establish charitable institutions for their relief would be reasonable as well as right. But the tendency to venereal indulgence is too strong to be repressed by such distant inconveniences, and the man or woman who failed to be restrained by other considerations, would not be so by the reflection that there were no Lock Hospitals.

The idea of preventing venereal indulgences and venereal disorders is perfectly ridiculous. But the community has a strong interest in mitigating an evil which cannot be averted, and in taking care that both its moral and its physical constitution suffer as little damage as possible:—ne quid detrimenti capiat res publica.

It is more easy to conceive than to describe the consequences of allowing syphilis to ravage the lower classes of society, without eleemosynary medical interference. Those ravages would speedily be frightful, and the worst forms of phagedæna would undoubtedly prevail to a desolating extent. It is not difficult to suppose that the evil would not stop here—infectious complaints of new descriptions would probably be generated—and the world might see a second time such a pestilence of lues as once carried death and terror on its wings. The disease originating in the lowest class would not long be confined to it, for sexual indulgences confound all distinctions, and a whore in the Haymarket not unfrequently poxes the son of a Peer. Those very persons who, from pious motives, reprobated the idea of pouring the oil of charity into the "foul" ulcer of the pauper, would be not unlikely to find in the mutilated bodies of their own children, the consequences of their overstrained scruples.

All practical people must come to the conclusion that the only mode of keeping down the nuisance of venereal diseases in the poor, of extending humanity to them, and, at the same time, protecting the community, is by institutions of the character of Lock Hospitals. We have already pointed out the disadvantages of foul wards in the General Hospitals. If the system of voluntary contributions shall prove, as we suspect it will, inadequate to maintain Lock Hospitals, the Government is imperatively bound to interfere, and the nation must save itself from the consequences of national infirmities.

We have hitherto looked at the question as one of morals and police. But it must be regarded in a scientific sense, and that a very important one. The disease is complicated in its phenomena, difficult to be treated, destructive when not treated well, and of every-day occurrence in all classes. Surely, then, medical men should enjoy some opportunities of seeing *en masse*, a malady which they will certainly be called upon to combat, and which will injure their patients materially if they do not thoroughly understand its management. This is a self-evident proposition. But, in reality, the very converse is acted on, and nine out of ten of those who go into practice have to learn all they *can* learn on the persons of the patients who are to come under their care. This may, for aught we know, be agreeable enough to those patients—the Public. Agreeable or not, it is the fact, for either there is no Venereal Hospital, or there are no pupils at it, or, if there are foul wards, the pupils are, for all useful purposes, excluded from them.

We have been tempted, by the importance of this subject, to say more on it than we at first intended. We return to what more strictly concerns us—the mode of administering mercury in syphilis.

### On the Administration of Mercury.

Dr. Colles devotes two chapters—fifty-one pages—of his book, to the examination of the question—how is mercury to be given so as to effect most good and least injury. A question of no little difficulty and no little consequence.

"It will be readily conceded," observes Dr. Colles, "that the efficacy of any remedy in the treatment of disease must in a great measure depend on the accurate knowledge which the practitioner possesses as to the most judicious mode of administering it ; this position, which is true in respect even to the most simple and innocuous medicine, will be found to apply with peculiar fitness to the use of mercury, when we reflect on the astonishing powers of this substance, its great superiority over every other medicine in the cure of certain forms of disease, the different results it is capable of producing, according to the mode in which it is administered, and, above all, when we consider that in the hands of the ignorant and the injudicious, it will not only fail to remove that complaint for which it had been prescribed, but may induce other diseases of a still more intractable nature, and that it even may, as too often has been the case, cause the sudden extinction of life." 25.

It is obvious, that, in order to treat syphilis by mercury with the best chances of success, it is necessary to be well acquainted with the phenomena of the first, and with the properties of the second. Until the surgeon has that acquaintance with both, he will assuredly do much mischief, and probably but little good.

Dr. Colles insists on the necessity of preparatory treatment prior to the commencement of a mercurial course. The older surgeons carried their precautionary measures farther than is usually thought necessary now.

"Those measures," observes our Author, "were not confined to plethoric and robust individuals labouring under primary symptoms, (such persons were soon reduced by bleeding, purging, warm bathing, and low diet,) but they also very judiciously extended the principle to those cases where the health appeared broken down, and where the venereal disease was accompanied by some morbid state of the system, not necessarily arising from that disease. At the time when I commenced the study of the surgical profession, this practice was not entirely extinct ; every venereal patient on his admission into hospital, was then, as a matter of course, ordered to be bled once, to be purged four times, and to have the warm bath twice in the first week, and not until all these directions had been complied with, was the use of mercury commenced. In the course of a little time the practice of bleeding was confined to those cases in which active inflammation attended on chancre or bubo, but still that of purging, warm bathing, and low living was in all instances strictly adhered to. In cases of secondary symptoms, in enfeebled and reduced habits, I never at that period saw any sort of preparatory process adopted." 26.

After some observations of a general character, Dr. Colles draws a picture of the folly and mischievousness of prescribing mercury because a sore is syphilitic, without inquiring what states accompany the sore and whether they are not likely to be aggravated by the remedy. It is indeed both lamentable and surprising to observe the want of discrimination too frequently evinced in the prescription of this medicine. If a surgeon believes a sore to be syphilitic he orders mercury as a matter of course, because it is the specific. That word specific has blinded him. Had mercury been an ordinary medicine, and given for an ordinary malady, he would have reflected on what its operation was, and whether that operation would be proper. But being a specific, that resolves all doubts, or rather it prevents any, and the common dictates of experience and good sense are no longer carefully consulted. Will not many of our readers confess with a sigh the truth of the following case?

"A young plethoric man," says our author, "who has been pursuing a dissipated course of life and pronounced by a surgeon to have a venereal ulcer and bubo, is directed to commence at once the use of mercury, either externally or internally: no previous measures are adopted, except possibly one dose of some cathartic medicine, no injunctions as to the necessity of rest and quietness, and confinement to the house, no alteration in his diet, in fact, no attempt to reduce the inflammatory and plethoric state of his system; but because he is young and healthy, and free from any of those diseases which appear in our nosological arrangements, he is therefore considered as in a fit state for the immediate use of this powerful medicine. Could I now induce such of my readers as have had practical experience, to recal to their recollection some of those cases of young men who have been thus hurried into a course of mercury, without any preliminary attention to their state of health, or without any particular instructions as to their mode of living; I have no doubt that every practitioner could adduce many instances in which their expectations of a safe and speedy cure have been disappointed; how frequently have they witnessed in such cases, that although the mercury acted on the salivary system, the ulcer assumed, not only a very unhealthy, but even a very novel and peculiar appearance, one which has sometimes caused them to doubt the correctness of their first opinion, as to its venereal character, and has induced them to lay aside the further use of mercury!" 29.

Both reason and observation may assure us that a disease like syphilis, evincing always a strong tendency to inflammatory complications, yet leading, like all specific poisons to a debilitated state of system, cannot be treated uniformly by any remedy of any sort. In the early stages, we must look for inflammation, and guard against its occurrence—in the later stages, we must expect debility, and adopt those means which will diminish it. According to the constitution of the individual the inflammatory tendencies will preponderate in one case, the weakness in another; while, in a third, the most troublesome case of all, we shall meet with that peculiar state, which we know as irritable, influencing remarkably local conditions, and requiring all the care and all the knowledge of the surgeon to contend with it.

Preparatory treatment we take to consist essentially in this:—the evil tendencies of a disorder, and the effects of the remedy adapted for it being known, to bring the constitution to that state which shall be best suited for diminishing the former, and for promoting the latter. In the instance of syphilis the first thing to be apprehended is inflammation, the second is debility. Preparatory measures, then, should be such as will tend to diminish inflammation, without unnecessarily debilitating the individual. In point of fact, it is from such preparatory measures that we have seen good, and such we invariably employ.

It appears to us as absurd to subject all kinds of persons with primary symptoms to one plan of preparatory as to one plan of continuous treatment. The means adopted by the older surgeons, of bleeding, sweating, purging every patient at stated times, are on a par with the directions of the physician in the play, "to vomit the North Ward, and to blister the South." This Procrustian methodus medendi is ridiculous in the eye of reason, and injurious in the hand of practice.

As different individuals have different habits and predispositions, and as syphilitic sores themselves vary in their characters and their inflammatory tendencies, the amount of preparatory depletion must also vary. It is for the surgeon to determine that amount in each particular case. But we would insist on this caution. He has before him a disorder which must run a certain and not a brief course—which must debilitate his patient more or less—and he has before him too a remedy which, however stimulating in some senses, must, in the long run, debilitate his patient also. Let him then husband the constitutional resources of the latter. If he draws an ounce of blood unnecessarily, its loss will be sensibly felt; not, perhaps, at the time of its abstraction, but still it will be felt.

We have seen a good deal of syphilitic complaints in all classes of society, and, in London at all events, we decidedly object to active depletion as a precautionary measure in the majority of cases. Aperient medicines, where the bowels are not irritable, diaphoretic salines, quiet, and moderate low living, are the means which we find adapted for the bulk of patients. These means pursued for a few days, in some cases more and in some less, are usually sufficient to reduce all inflammatory disposition, and to pave the way for the mercurial course. The local applications should correspond with the general treatment. While we open the bowels and prescribe salines, we order diluted black-wash, or even a bread and water poultice for the sore. It is rarely that these measures are not adequate for the prevention of any untoward phenomena.

Suppose a patient properly prepared, the sore uninflamed, the secretions in good order, the system in a tranquil state. He is to be put on a course of mercury. What are we to expect from it—how do we intend to exhibit it? This is a matter of some consequence. Let us turn to Dr. Colles. He reasons thus:—

"When mercury is exhibited for the cure of any other disease, as well as for Syphilis, we shall find that its sanitary impression on the disease is contemporaneous with its action on the salivary system, and that when the latter effect has not been produced, neither will the former have occurred. Thus in cases of acute inflammation of a joint, or of the dense membranes of the eye, we find that the progress of the disease is arrested the moment the salivary system becomes affected; and even in cases of other diseases, which cannot be considered as purely inflammatory or acute, the same remark will be found to hold good: thus in cases of orthopnœa depending on disease of the heart, with effusion into some of the thoracic cavities, and in which we commonly prescribe mercury in combination with squill and digitalis, the patient is not at first sensible of any improvement, but almost invariably, as soon as the gums become affected, he experiences relief, and perhaps the very next morning after this occurrence he tells us with joy and gratitude that he is considerably better, that he has passed a night of refreshing sleep, and that he has been able to do what he could not have done for weeks previously, namely, rest in the recumbent posture without any of that distressing and alarming sense of suffocation under which he had previously laboured, and which always supervened the moment he sunk into that position. It is unnecessary to particularize many other diseases in which the same fact occurs; indeed it may be asserted as the general rule. The contrary also will be found equally true: that is, that mercury will not prove serviceable in any disease for whose cure it has been prescribed when it does not produce its wonted effect on the salivary system. How often has this been verified during that time, when it was the fashionable practice to prescribe a course of mercury in all chronic affections of the liver? It then happened over and over again, that slight delicate females have been subjected to this treatment; friction perhaps has first been tried, and this failing to relieve the complaint, (because it had also failed to affect the salivary system,) the internal use of mercury has then been substituted, or probably combined with the former; thus the medicine has been persevered in, and the doses increased, even to an extravagant degree, but yet withal no salutary effect has been induced; on the contrary, the little remnant of strength has suffered so materially, that at length the mercury had been laid aside, and the friends, as well as the medical attendant of the patient, have had reason to express, not only their disappointment, but also their amazement at the inefficacy of the mercury, of which the patient had taken fully as much as would have sufficed to salivate at least half-a-dozen young and vigorous men. If then it be so very generally found, that whenever mercury exercises a salutary influence over disease, it at the same time always affects the salivary organs; and if again whenever it fail to produce this latter effect, it be also found altogether inoperative in the cure of disease, it is surely a fair and legitimate conclusion to affirm that ptyalism marks the natural and salutary operation of this mineral." 33.

To a certain extent these propositions may be granted, and they may and do constitute an à priori reason for supposing that salivation is as requisite for the cure of syphilis, as for the cure of other maladies which mercury actually does cure. Dr. Colles proceeds to apply the analogy, and in some degree to qualify it.

"Let it not however be inferred from the foregoing statement, that I would wish to measure the efficacy of mercury by the amount of salivation which it excites; on the contrary, the degree of ptyalism that I am always anxious to attain, is merely an increased secretion of saliva, accompanied by swelling and superficial ulceration of the gums, and sometimes also of portions of the lining membrane of the cheeks and lips; this I am desirous of attaining as a sort of index which denotes first, that the mercury is acting in a safe and salutary mode upon the system; and, secondly, that it displays that degree of power or energy of action, which will be sufficient to eradicate the disease: so certain do I feel of the correctness of this view, that during a course of mercury for the cure of Syphilis, should this ptyalism be suffered to decline for some days, I should fear that all the additional mercury which may have been subsequently given in this more feeble manner would prove useless. that is, unequal in the cure of disease." 33.

However specious the analogy between syphilis and other maladies curable by salivation, it is clear that experiment and experience only can really decide the point. We cannot agree with Dr. Colles. We do not find it necessary to produce ulceration of the gums, nor do we believe that any quantity of mercury *minus* that which occasions this ptyalism must prove useless. This is a matter of fact, not of reasoning—of observation, not of hypothesis.

Salivation being an evil, it is of course an object to dispense with it if possible. All surgeons, in the present day, concur in admitting that salivation to any amount is unnecessary. It cannot, in the moderate way in which it *is* employed, do much in removing disease, but is rather an indication that the constitution is under the influence of the medicine. If this be so, why should we suppose that a quantity of mercury, insufficient to occasion salivation, but continued for a longer time than when salivation is effected, exerts no influence on syphilis? We see no valid reason for that supposition; and so far as our observation has extended, it has countenanced the idea that mercury given short of salivation does cure the disease.

We do not wish to be misunderstood. We do not recommend very small doses of mercury; but we think, that if this medicine is given to the extent of making the gums a little sore and spongy, and of slightly increasing the salivary secretion, and if by the continued administration of mercury *that* effect is kept up for a certain length of time, a reasonable security against secondary symptoms is obtained, with very little detriment to the general health of the patient.

We see a considerable number of patients affected with primary syphilis. We never purposely *salivate* them; yet we very rarely indeed find secondary symptoms follow. If we may venture to draw a comparison, which will appear to be tinctured with vanity, and must be surrounded with uncertainty, we should say that we have fewer cases of relapses than most of the surgeons with whose practice we have become acquainted. But although we do not salivate, we take care to submit the patient to the mercurial influence for never less than five weeks, and frequently for a longer period; and we take care also to see that no induration, nor any morbid appearance, is left in the cicatrix.*

The regimen of patients during a mercurial course is a subject of considerable importance. In former times, as Dr. Colles observes, it was the custom to have the patient covered with a thick flannel, or woollen cloth dress, and the head even covered with a sort of hood, so that the face only was exposed; he was also confined to a very warm, close room, from which the external air was most carefully excluded. The consequences of such a system were prominently shewn in the furious salivations, the emaciated figures, the pallid looks, the occasional mercurial erethismus, the still more frequent cachexia, which patients formerly displayed.

Mr. Hunter was instrumental in changing the system; but, as he contended that diet exercised no influence at all on the specific action of mer-

* There are some sores attended with a degree of induration, which no reasonable quantity of mercury will remove. But such instances are the exception, not the rule.

cury, he only substituted one error for another. Still the world is deeply indebted to him; for the error he has introduced is incomparably less mischievous than the one which he exploded. On this point we quite dissent from Dr. Colles, who conceives that the system of our ancestors was the better one. "The proof of the pudding is i' the eating," and the proof of the present system being the better one, is the acknowledged diminution of those terrible cases which were formerly so rife. That diminution must in fairness be ascribed in part to the alteration in the regimen and management of patients, as well as to the alteration in the mode of exhibiting mercury.

Our readers must not, however, suppose that Dr. Colles is an advocate for acting on the wisdom of our ancestors in this case. He admires, but does not literally comply with its precepts.

"Should our patient," he remarks, "appear debilitated after the moderate and the desirable degree of ptyalism has been induced, then we may safely allow him a full and generous diet, from which, however, I would still exclude all stimulating food or drink; moreover, if about this period the patient's strength fail, or if the local symptoms do not improve as we had expected, we may then combine with great advantage bark and opium with the mercury, while at the same time we should reduce the doses of the latter." 35.

Dr. Colles, from his time of life as well as his position, has necessarily seen much. Not only has he witnessed a good deal of the venereal disease, but he has witnessed also great revolutions in the treatment of it, and can tell us of bye-gone times. The following quotation, though lengthened, will interest our readers.

"When I call to my recollection the results of my own observations on the treatment of this disease, made thirty years ago, and compare it with that of the present day, I think the comparison speaks decidedly in favour of the plan which was then pursued. For some years after I entered on the study of the profession, a surgeon felt himself rather humbled if he allowed a venereal bubo to suppurate; and if secondary symptoms appeared, he was considered to have mismanaged the case, and not unfrequently lost for ever after the total confidence of his patient. It is true, that at that time mercury was often used in excessive and in dangerous doses, salivations most profuse were excited, and which were attended with all their accompanying evils; but still, on the other hand, the patient who escaped these perils was generally freed at once from the disease. The regimen then was as strict as the medical treatment was severe. Lodging-houses were established in Dublin, solely for the reception of young gentlemen who required to go through a course of mercury; and these houses were always fully occupied—so seldom was it that a young man could, while living among his own family, undergo the severe discipline to which the surgeons of that day thought it necessary to subject him.

But from the time that Mr. Hunter's work on the venereal disease came to be generally read and acted upon by the surgeons of this city, the discipline became not only less severe, but actually as lax as Mr. Hunter himself could wish. Young men, pleased at the removal of these restraints, too frequently overstepped even the moderate bounds which were prescribed for them; and surgeons, finding that secondary symptoms frequently appeared in cases so treated, were glad to adduce Mr. Hunter's theoretical opinions of 'disposition to diseased action,' and so forth, partly to justify themselves, and partly to satisfy their patients; so that at length, from this, and perhaps from other causes, which I shall not now consider, those cases of primary venereal disease, which, under the old practice, required six or seven weeks for their cure, were, under the new plan of treatment, found to require as many months, or even years. By the former, the disease was really and quickly cured; but, by the latter, it is only pursued from one resting-place to another, so that the patient's mind is often kept in a state of suspense and anxiety for very many months,—the symptoms disappearing for a few weeks, and then returning; and so this scene may occur over and over again, until, by some lucky chance in the treatment, or in the effects of the medicine, the system shall come to be finally relieved from the disease.

While I freely admit that excessive over-doses of mercury have inflicted the most severe evils, yet I as confidently affirm that within the last twenty years, the same medicine, when employed in under-doses, or rather when it has not been pushed so as to induce its legitimate action, has been productive of infinitely more mischief; and I feel confident that this position could be supported both by professional as well as non-professional persons were the effects of the latter practice as obvious and striking as those of the former; but they cannot be so fully appreciated by the ignorant, and they have not been sufficiently attended to by the profession." 37.

Of course the recollection of facts must be received with deference. But it must be owned that the recollection of facts, particularly when comparison is exercised, is apt unconsciously to be warped by speculative opinions. Under the influence of those opinions, we look at all objects through a mist, and, against our will and without our knowledge, they are distorted by the medium through which they are regarded.

We find it difficult to reconcile the reminiscences of Dr. Colles with general experience and common belief. It is now almost a canon of faith that, in consequence of the mitigation of the mercurial code, venereal cases have lost much of their severity, and that fewer victims are immolated at the shrine of syphilis. But Dr. Colles reverses the idea, and pronounces boldly that the contrary is the fact.

It is evident that the point is not to be decided by argument. It is essentially statistic. The elements of calculation not being before us, we shall not attempt to dogmatize on the results. But perhaps one or two observations may help the reader in arriving at an accurate estimate.

Dr. Colles contends, that if too much mercury was given formerly, too little is given now. He necessarily speaks from personal observation. It may be, and it probably is the case, that Dr. Colles has seen worse practice than ordinary in this complaint. We are induced to think so partly on account of the nature of the assertion itself, so contrary to all that we have observed, so contrary indeed to general observation—and partly because Dr. Colles has practised in Dublin, where Mr. Carmichael's views have been promulgated, and where they must exert considerable influence.

We readily grant that the mass of medical men have no fixed principles of treatment in regard to syphilis. They give mercury for a disease with which they are so imperfectly acquainted, that whether *that* goes right or wrong, they are equally at a loss for any criterion by which to regulate *its* use. The consequence is, that we daily see instances of mercury given for sores which it is aggravating, and discontinued when its cessation will almost certainly entail secondary symptoms on the patient. If *we* see this in London, Dr. Colles is more likely to see it in Ireland, on account of what we have already stated—the influence of Mr. Carmichael's doctrines.

It appears to us then, that a comparison between the past and present practice will differ with every individual who institutes it. As circumstances are or have been, he will find the one plan or the other more successful—a well-informed mercurialist of former days having been, we dare say, both a better and a luckier surgeon than an ill-informed practitioner of the present. In the following sentiment we coincide entirely with our author :—

"No doubt, observation, experience, and sound judgment are very requisite to enable the practitioner to decide whether those changes which appear at the critical moment when mercury is about to excite ptyalism be really unfavourable and alarming, or whether they may be merely the natural effects of the mercurial action; this, however, is not the only instance which the practice of surgery presents, in which accurate observation, discrimination, and judgment are required to practise the profession with safety and success; these are qualifications which can only be attained by long and attentive experience; no precise rules, therefore, can be laid down to guide the surgeon at this critical juncture, neither can any words impart to him that practical knowledge, without which he cannot conduct the further treatment of the case with satisfaction to himself, or with true advantage to his patient." 39.

This is perfectly true. It is impossible to make what is called a practical man by books; nay, it is impossible to communicate any but the grosser part, the mere results, of practical knowledge. This consists in a quick observation and a nice discrimination of particulars—in an accurate and rapid calculation of probabilities—in a just application of those remedies to particular states, the applicability of which to those states has only been learnt by experience. We might as well attempt to describe all the tints in an Autumnal evening sky, all the hues of an Autumnal foliage, as to convey by words genuine practical information. In those who have it, it appears almost intuition; in those who have it not, the deficiency is too readily perceptible, and that even by common eyes. All that can be done by one who professes to instruct others, is to put them on the best method of study—to correct errors of principle—and to convey, as distinctly as possible, the great general results of practical experience.

This brings us to the termination of one, and the commencement of another chapter, in our author's book. The one on which we enter is appropriated to the examination of the methods of administering mercury—a subject of no trivial consequence to the practical surgeon or physician.

### ON THE METHODS OF ADMINISTERING MERCURY.

Dr. Colles supposes that his patient is a young man, in good general health, affected with primary venereal symptoms. In such a patient, he is anxious to effect that ptyalism which our readers have learnt that he deems essential. He first speaks of the employment of *mercurial friction.*

This is more resorted to in hospital than in private practice, for obvious reasons. Patients object to the *bore* of it. There can be no doubt that it is a very valuable, perhaps the most valuable, mode of introducing mercury into the system in cases of venereal disease. It may be either a very active or a very mild method—adapted for severe, or for slight cases—and open to scarcely any objection except that which is founded on the trouble it occasions. Slight as that objection may appear in the eye of reason, it has nearly banished mercurial inunction from private practice in London. For ourselves we must say, that although we should wish to employ it often, we very seldom have recourse to it. As it is a powerful and a very manageable remedy, all surgeons should know *how* to manage it. Dr. Colles' being essentially a practical book we shall lay his plan before our readers.

"The patient should be apprised of the necessity of rubbing in each dose of the ointment carefully, but not violently, and this he should do in the morning rather than at night, and for these reasons; first, the skin is soft in the morning and will bear friction better; and secondly the sleep will not be postponed or disturbed, as usually happens when the nightly friction is employed, the patient being fatigued thereby, and thrown into a state of febrile excitement which is inimical to sound repose.

As to the mode of rubbing in the ointment, the patient should be directed to divide the whole quantity to be used into four parts, and then rub in each portion perfectly and snccessively, until all are consumed: it is better to apply it to one limb only on each day, as thus that pustular eruption, which is a common consequence of mercurial friction, is less likely to be excited. I prefer making the patient apply the ointment himself, whenever his strength will admit of the exertion, because the friction of his own hand is less uncomfortable, and less likely to produce irritation and eruption, than that of any other person, even though protected by a bladder ever so well prepared.

I do not advise the thighs to be shaved, as many surgeons do, because in a few days, when the hairs grow, and become stiff, more irritation is produced, and thus the tendency to pustular eruption is increased.

During the course of mercurial friction, the patient should wear the same drawers both by day and night; he will thus have some portion of the ointment constantly applied to the surface; some of which will probably be absorbed during the intervals between each friction. When the same part of the body has been rubbed two or three times, it is advisable to wash off the remains of the ointment with warm water and soap, and this ablution should be made the night before the next friction.

When we deem it necessary to employ mercurial friction with a patient who is in a very weak state, or more especially with one who is in a constantly feverish condition, we must direct the friction to be performed by some other person, to whom we should give the following instructions:—The servant should be furnished with a pig's bladder, which, after having been well steeped in warm water should be turned inside out, then well impregnated and softened with sweet oil or fresh lard. After this preparation, it is to be tied round the wrist of the assistant, and the ointment rubbed by him according to the directions already given." 42.

The dose for each friction is in Dr. Colles's practice ʒss. If he is anxious to induce ptyalism rather early, he orders, at the same time, five grains of blue-pill to be taken every night at bed-time.

It must be admitted that this is very moderate treatment. We do not consider ourselves as violent mercurialists—the whole character of our practice, and, we think, the tenor of our observations, are the reverse of that. Yet, when treating primary syphilis in a healthy person by inunction we do not hesitate to order ʒj. to be rubbed in daily. Of course the continuance of the quantity hinges on the effect produced upon the patient.

Upon this point, we may quote a passage from Mr. Hunter's work.

"If it, (mercury), is given in very small quantities, and increased gradually so as to steal insensibly on the constitution, its visible effects are less, and it is hardly conceivable how much may at last be thrown in without having any visible effect at all."*

If mercury is exhibited internally, Dr. Colles, to whom we return, prescribes five grains of blue-pill twice daily—a very judicious dose. If calomel is, for any reason, preferable, he gives two grains at bed-time. This we think rather under the mark. If purging is to be avoided, the mercury should be combined with a small quantity of some opiate.

With respect to the forms of mercury here mentioned we would venture to make these remarks. The blue-pill is the milder form, and suits most constitutions. If it does not gripe nor purge, there is no reason why an opiate should be added to it. If it does gripe or purge, opium, in a quantity varying from $\frac{1}{12}$th of a grain to one grain or more should be combined with each five grain blue pill. If the latter, however, guarded purges, the mercurial inunction should be used. An occasional aromatic rhubarb draught frequently prevents the blue-pill from griping or purging.

Calomel seems best adapted for strong habits and inflammatory cases. In the latter it should be combined with purgatives, or with opium according to circumstances. As a general rule, calomel should be avoided in the treatment of primary syphilis. Yet a dose or two of calomel and James's powder, followed by saline aperients, is a very good introduction to a mercurial course, whether of inunction or blue-pill. Such are the results of our experience.

About the fourth day, remarks Dr. Colles, we usually find, on *interrogating* the patient, that a metallic taste in the mouth, and other symptoms indicate the coming ptyalism. About the sixth or seventh day, this is usually established. Dr. Colles insists on the surgeon paying the most strict attention to the patient from the third to the seventh day. It is at this period, he observes, that the attentive and judicious surgeon can be of essential service by giving a right direction to the medicine, as well as by counteracting any injurious effect it may produce. Thus during this critical period, the patient is liable to attacks of griping, frequent desire to go to stool, and tenesmus; these efforts are attended with only slight evacuations, which chiefly consist of mucus tinged with blood ; sickness of stomach and vomiting also often supervene, the skin is hot and the pulse quick ; all which phenomena are explained by the fact that the specific influence of the mercury has taken effect upon the alimentary canal instead of the salivary system. This dysenteric affection so generally appears at this period that the patient should be forewarned and prepared for it. He should be directed to discontinue the use of the mercury, as soon as he feels this uncomfortable effect, and he should be provided with draughts, containing each Tinct. Rhei. ʒj. Tinct. Opii gutt. xx. in any appropriate vehicle ; one to be taken after each dysenteric stool. An opiate enema may be used instead of the draught, whenever the stomach rejects the latter. A gentle diaphoresis also should be encouraged by the tepid bath, or by bathing the feet in warm water. In the course of a day or

* "To give an idea of this, ten grains of the ointment used every day, during ten days, affected a gentleman's mouth. The ointment was of equal parts of mercury and hog's-lard. But, by means of omitting the ointment occasionally, and returning to the use of it, he at last rubbed in eighty grains every night for a month, without having his mouth, or any of the secretions, visibly affected."

two this febrile excitement will, under this plan of treatment, have somewhat subsided, the bowels will remain free from disturbance, and then we shall generally find that the mouth has become a little more affected ; and should we wish to have it still more so, we may resume the use of the mercury, and continue it in such doses as the circumstance of the case may require. Dr. Colles goes on to remark, that, sometimes the mercury displays its effects in a somewhat different manner. The patient has fœtor and soreness of the gums, which ulcerate more than they should do, but he has no augmented flow of saliva. In such cases there is usually too much fever. If the mercury is continued, there is more severe ulceration, and perhaps the fever becomes alarming. This is what the lower order of the Irish call "a dry course." It does not cure the disease. It may, however, says our author, be converted into the full and legitimate ptyalism by reducing the doses and lengthening the intervals between them, and at the same time using such means as are calculated to reduce the rather high degree of fever which attends this peculiar condition of the mouth.

In a few patients, the mercury affects the throat and not the gums. Such persons complain, about the fifth or sixth day, of a sore throat.

"On inspecting the fauces, we discover a degree of erysipelatous blush on the arches of the palate, and some inflammatory thickening of the velum palati : on the tonsil, and generally at its upper extremity, we see a superficial ash-coloured slough ; one side only may be thus affected, or both may be engaged in it. I need hardly say, that in such a case a further persistence in the full doses of mercury would not only prove ineffectual for the relief of the venereal symptoms, but would also be attended with considerable danger to life, by inducing a sloughing condition of the fauces." 45.

Mercury, in such a case, must be withheld or much diminished in dose. The inflammatory condition of the throat must be set at rest, and the mercury then resumed. It may or it may not be borne well. The patient requires to be carefully watched.

Such are the sentiments of Dr. Colles on this important subject. So far as our own observation has extended we should say, that, in the main, it coincides with that of Dr. Colles. The practical rules at which our experience has enabled us to arrive, are these :—

1. To prepare a patient for a mercurial course, by low living, smart purging, preventing exposure to wet and cold, and mild local applications.

2. To enjoin during the course, moderate and regular, but not very low living—little exercise, and that gentle—the sedulous avoidance of wet, cold, and night air.

3. To make a point of watching the patient closely, and insist on his presenting himself every three or four days. If pyrexia occurs, no matter what its cause, to prescribe such remedies as are calculated to remove it, and to diminish or suspend the mercury until the pyrexia has subsided. We are sure that this is highly important, for either fever or local inflammation is a primâ facie reason for discontinuing the employment of mercury as a specific. It may in some cases be advantageously combined with diaphoretics, or with purgatives, but in others it must be abandoned, during the continuance of the inflammatory state.

4. To observe whether the patient loses flesh and becomes lowered, and if he does, to give sarsaparilla, or some mild tonic, with the mercury, and to allow a more generous regimen. This is a point of great importance. Some persons if once allowed to be much pulled down by the mercurial course, are scarcely able to shake off its consequences. They continue pale and thin, and out of health for a long period, and not unfrequently, phthisis, or some malady indicative of constitutional debility, silently steals on, and carries them off.

5. On the whole, we would sum up what we have learnt in a few words:—to ensure a *full* mercurial course—to discontinue or modify mercurial remedies if inflammation or fever is set up—to maintain the general health. The details of such treatment will vary with the nature of the case, will be regulated by the judgment of the surgeon, and will materially influence the fate of the patient.

We cannot say that we attach so much importance as Dr. Colles seems to do, to the precise time at which ptyalism is induced. Nay, we are not so impressed as he is with the necessity for ptyalism at all. It is true that his notions of ptyalism are moderate, and it is true also that the quantity of mercury he gives is rather under than over what we prescribe. Substantially, then, we agree, for neither he nor we think the patient secure with a less efficient course than we employ. But, still, we repeat that we suspect there is a little hypothesis in our author's minor opinions. Take the following passage as an instance.

"I have been thus particular in stating at what period the surgeon should wish ptyalism to commence, when using mercury for the cure of primary symptoms, because I am firmly convinced, that he cannot count upon a cure, if the salivation occur at a period much different from that above-mentioned; for should it be suddenly excited, and even though not very profuse, it will yet leave the disease uncured: perhaps the primary symptoms may be removed by it,—oftentimes they are not; but at all events the secondary symptoms will not fail to make their appearance, sometimes in full vigour, though often under a more subdued form. This I have seen repeatedly exemplified in cases of iritis, treated by calomel alone, or by calomel combined with frictions. I have also repeatedly seen mercurial fumigations which were used to stop a destructive ulceration of the throat, produce a speedy and profuse salivation, by which the ulceration has generally been stopped, but in almost every such instance the other venereal symptoms were only checked for the moment, to return with renewed vigour. And here I must remark, that after such sudden and profuse salivations, I have uniformly found the symptoms were very unmanageable, and by no means yielding in the usual way to a subsequent use of mercury.

On the other hand, if the salivation be very late in appearing, not only will the patient have to lament the loss of so much valuable time, but the surgeon will too frequently find that the system of his patient has been so irritated by this protracted, though moderate use of mercury, that the ptyalism excited at this late period, is not borne with the same ease by the constitution, nor is it productive of the same improvement in the symptoms that would have resulted from it, had it come on at an early period of the treatment." 47.

We must say that we have again and again seen patients undergo a mercurial course, and exhibit no ptyalism at all worth speaking of, without any secondary symptoms ensuing. We have seen this so often, that we care little about the occurrence of ptyalism, provided a certain quantity of mercury is taken for a certain time, and that certain other effects are discernible.* If

* The removal of the induration of the cicatrix, for example.

this be true, and our senses seem to tell us that it is, we cannot go all the way with Dr. Colles.

But much that he tells us is strictly correct, and as important as correct. Profuse salivations, rapidly excited, have under our observation been followed by such consequences as Dr. Colles mentions. Many of the miserable cases of cachexia which we witness have had their origin in salivations of this description. But to proceed.

When the desirable action of mercury is once obtained, it is easily kept up in the majority of instances. We quite agree with Dr. Colles, that it is better to prescribe too long than too short a course. If *the general health is maintained*, and it may be so, we never saw any mischief arise from the *very long-continued* use of mercury. But we have seen (and who has not?) many and many a case of secondary symptoms after a very short course.

If the mercurial action sinks too low, Dr. Colles has fonnd it most advantageous to give pretty smart doses of calomel, viz. three grains, with one of capsicum, two or three times a day, at the same time that the former doses of the ointment are resumed.

Dr. Colles next examines the untoward circumstances which occasionally present themselves during a mercurial course. It is always well to ascertain from a patient if he has undergone a mercurial course before, and, if he has, whether it was attended with peculiar effects.

A. Some patients are salivated by very small quantities, even by single doses of mercury. Such an event is to be deprecated. If it occurs, or if it threatens, the patient should be much in the open air, live generously, and take a diminished dose of the mercurial, at more distant intervals. The remedy must be insinuated into the system. If judiciously managed, such persons will frequently bear sufficient quantities of mercury at last.

B. In some persons, the ordinary quantity of mercury produces too trivial an effect. The quantity of the medicine must be increased. This requires great judgment on the patient's part, and no precise rules can be laid down. The following are Dr. Colles' views upon the subject—one of no slight moment.

When, he says, the dose is to be increased, it certainly ought not to be less than one half, and sometimes it may be doubled at once; the former proportion will probably be the better. If the patient has been employing mercury both, by friction and internally, the dose of both should be increased, but if he has been using friction alone, then this should be continued at an increase of one-half, and he should take in addition five grains of the blue-pill every night. If in such cases we did not *early* increase the doses, the system would become familiarised to the mercury, and at a late period we should employ very large doses, and thus run the risk of exciting a profuse and dangerous salivation.

The plan pursued in such a case, one in which the patient's future safety is at stake, will vary with the speculative views of the surgeon. If, with Dr. Colles, he thinks salivation indispensable, he will augment the dose of mercury, until that is obtained, or so far as it is prudent to proceed with the object of obtaining it. If, with ourselves, he looks for salivation with less anxious anticipation, he will be content with the manifestation of other effects of the remedy, and with the conviction that his patient is taking that quantity which he knows by experience usually cures.

We would remark that, whatever be the views of the surgeon, he has always the means of increasing the action of mercury, by augmenting the temperature in which the patient is kept, and by lowering his constitutional powers. A certain dose of mercury may exert no perceptible influence on a man who is eating meat daily and drinking porter or wine. But confine that same man to slops, make him use the warm-bath, and probably a smaller dose of mercury will salivate him.

"We sometimes find in other constitutions, that two or three doses of mercury induce a feverish state of the system, without the peculiar fœtor of the breath, or any of those other symptoms that indicate the approach of ptyalism: the skin will be hot, the pulse quick, with great general restlessness; under these circumstances, we infer that the mercury is disagreeing with the system, and should we now either continue its use, or increaee the dose, we shall only add tothe mischief by increasing fever; and if we still persevere, we may endanger the life of the patient; in general however, he is soon reduced to such extreme weakness, and is so sensible of becoming daily worse, that he will himself oppose any further use of the medicine. Whenever this febrile state of the system, unaccompanied by any sign of its acting on the salivary organs is thus early induced, we should at once desist from the further use of mercury, and endeavour to allay the fever by exposure to cold air, and by the adoption of such other means as may be best suited to the circumstances of each case. Having accomplished this, we must allow some time for the recovery of strength, before we venture to resume the mercury; and when we do so, we should begin with smaller does, and these at longer intervals, for experience has shewn us that the former quantities were such as overpowered the system. We must also endeavour to obviate any febrile action by occasional purging, by tepid bathing, and by the frequent use of mild diaphoretics; we are now to proceed cautiously, increasing the strength and frequency of the mercurial doses, until we gain the desired object, namely, a genuine and wholesome salivation. Here I would observe that this difficulty is not an uncommon consequence of plunging a healthy vigorous patient into a course of mercury without any previous preparation." 53.

All this is so practical, so judicious, that we have not a word to add to it.

D. Not unfrequently individuals resist obstinately the effects of mercury without having any fever induced. The sore pursues its course apparently uninfluenced. Under these circumstances, the mercury may be discontinued, or, as is usually the case, the dose of it may be increased. Dr. Colles has known two drachms, and even half an ounce of ointment, ordered to be rubbed in each night and morning, and at the same time large doses of Pil. Hydrarg. Calomel, or Hydr. Calcinat., given internally for some days; the result of this practice has been, that in some cases it has produced that general weakness and that tendency to faintness which mark the approach of erethismus. Then, of course, the remedy is abandoned.

In some instances, these excessive doses induce a violent ptyalism. We need not picture *that*. Some of our readers may have seen, all may conceive it. Such ptyalism may succeed in curing the disease for which it was excited, and which it almost exceeds in evil, or it may leave the unhappy patient with his original malady still afflicting him—a miserable position.

E. Dr. Colles considers the method of avoiding the various ills that have been mentioned.

If we ascertain that, on former occasions, the patient has been nearly insensible to the action of mercury, we should begin, says our author, with small doses, at longer intervals than usual; or, if we have commenced with the ordinary doses, we should not persevere with them, but should purge the patient repeatedly, enjoin low diet, and prescribe the frequent use of the warm bath.

"It has sometimes happened that, during the employment of these last mentioned means, a safe and mild ptyalism has supervened a few days after the mercury has been laid aside. This apparently anomalous fact is no weak proof that the cause of our failing to produce the desired effect in the first instance, was owing to the mercury having been administered in an injudicious, and as it were in too forcible a manner. But if with this treatment, no signs of ptyalism should appear, we must still persevere in the same constitutional means, until we conceive that the system is totally free from all mercurial impregnation; to induce this state may occupy twelve or fifteen days. We then commence a new course of mercury, and, taught by our former experience, that full doses of the medicine failed to produce the desired effects, we now direct it in very small doses, but at the same time we persevere in the same constitutional remedies as before; after a few days passed in this way we are sometimes pleased to find the signs of an approaching salivation gradually advancing in the very manner we are so desirous to effect. It appears to me, that this very precept which has been just inculcated, is the great secret which will serve to guide us in the management of those cases which have hitherto often excited great uneasiness in the mind of the surgeon; and it is a remarkable fact that this obstinate state of the system to admit of the influence of mercury, is not unfrequently met with in those very patients, who on former occasions had been salivated rather too quickly with the ordinary doses of mercury, and that too by the very same surgeon who is now so baffled in his attempts to produce the same effect. If surgeons of much practical experience will endeavour to recal to their recollection all the circumstances of such cases, I believe they will find, that on the latter occasions, in which they had to treat such cases, they were very anxious that the effects of the mercury should be quickly established; now, in my opinion, this very anxiety, by inducing the surgeon to urge the mercury too freely, has been one source of its failure, and of the disappointment of his surgeon." 57.

There can be no doubt of the value of the preceding practical precepts; but we are not so sure of the soundness of the reasoning. Dr. Colles says, that the larger doses of mercury fail to affect the system, because they are given in too forcible a manner. It appears to us that the smaller doses do the business, not because they are smaller, but because they are conjoined with purgatives, low diet, hot baths, &c., means well calculated for promoting absorption, and for quickening the action of any medicine. Were the same large doses of mercury conjoined with the same constitutional treatment, the effect of the former would not, we fancy, be less, but greater. This, however, is a speculative question. We quite coincide with our author in his practical directions.

F. Dr. Colles has met with eight or ten individuals on whom mercury did not act as a sialagogue at all. Yet their venereal complaints were cured. This rather bears against his opinions respecting the necessity for ptyalism.

G. The general directions given to patients by Dr. Colles are to avoid exposure to wet or damp, to cold, or to night air. After a mercurial course, persons are subject to rheumatic pains; they generally too grow corpulent.

Dr. Colles takes no notice of a tendency to jaundice after a mercurial course. Yet we have seen several examples of it. The reason would appear to be, that the liver having long been stimulated by the mercury, and that stimulus being discontinued, is liable to perform its functions irregularly. We always recommend patients to regulate their bowels very carefully after the termination of the course.

H. Dr. Colles makes some lengthened observations on the mercurial eczema. His descriptions are accurate, his precepts judicious; but all that requires notice is the advice he offers on the treatment of those persons, (fortunately few), who evince a strong disposition to suffer from the eruption on taking small doses of the medicine. The plan, he says, to pursue is this: in addition to the purging and warm bathing, which have been alluded to before as a judicious preparatory course, we should enjoin the patient to wear lighter clothing than usual, to exercise in the open air, during the greater part of the day, to keep the windows of his sitting-room pretty constantly open, to keep on light covering at night, to live abstemiously, and on food of the least stimulating quality. The mercury should be administered at first in extremely small doses, and at long intervals; by degrees the former may be increased, and the latter shortened, in proportion as we find the medicine agree with the system.

I. "We should carefully distinguish between erythema mercuriale and another, but more partial, eruption arising from the use of mercury. They both come on under similar circumstances; both seem to be excited by the first impression of mercury on the general system. Our attention is attracted to this latter eruption by our patient informing us that he fears he has got the itch, that he could scarcely get a wink of sleep for one or more nights preceding. He then exhibits on his hands and wrists an eruption beginning with small but very distinct red papulæ, some of which, in a more advanced stage, have vesicles on their apices; they chiefly occupy the anterior surface of each wrist, and of the fore-arm half way up to the elbow; the backs of the hands and fingers are also thickly beset with them. On first view, this eruption closely resembles a form of itch, in which the vesicles are small; but on more careful examination you discover that the clefts between the fingers are altogether free from the former, while they are known to be the principal seat of the latter. This eruption is accompanied by a slight degree of fever, and generally by marks of commencing ptyalism.

I cannot say what changes or effects on this eruption would be produced by persevering in the use of mercury, because all the patients in whom I witnessed this symptom were also affected with a smart degree of fever, and complained so bitterly of the itching and of the restlessness caused by it, that I felt afraid to go on with the mercury until the irritation of this eruption had subsided. A few days use of the antiphlogistic regimen, and abstinence from mercury at the same time, were sufficient for the desquamation of the pustules, and the removal of this rare effect of the mineral." 67.

K. "Another effect of mercury, allied perhaps to the foregoing, which the surgeon should watch for, is an excoriation of the skin on the corresponding surfaces of the scrotum and thighs. If this be discovered in its commencement, it will be seen as an excoriation in the very angle between the thigh and scrotum; from this it spreads over the entire extent of the opposed integument of these parts, and a profuse discharge of a very fætid nature takes place." 68.

Dr. Colles goes on to observe, that, in addition to the parts already mentioned, this disease will also affect the skin in the vicinity of the anus, where the integuments of the nates lie in contact with each other. Occasionally, too, we meet with instances where this disease affects only the skin in the vicinity of the anus, while the scrotum and adjacent skin of the thigh remain perfectly free.

The duration of this affection, says our author, varies from eight to fifteen or twenty days. For the relief of all these sufferings we find opium unavailing; it fails to procure any continued sleep. The antiphlogistic regimen scarcely moderates the fever. The most immediate and most effectual relief is procured by local means; the most efficient of which is dusting the excoriated parts with equal parts of lapis calaminaris and starch, very finely levigated: this is to be laid on pretty thickly, and then a fold of old linen interposed between the two adjacent skins. Another application, which often procures immediate ease, is the black wash of aq. calcis and calomel; the affected parts are to be kept asunder by lint constantly moistened with this lotion.

We must confess that we have never seen this affection as a consequence of mercury, though we have seen it several times as a venereal symptom. Dr. Colles offers no sort of evidence of its mercurial origin, indeed the only case he relates rather tends to disprove such an opinion, and an observation which he offers tends also the same way. The case we need not quote—the observation we may:—"a repetition of the same forms and doses of mercury which are supposed to have given rise to the excoriation, may, when the excoriation is once healed, be resorted to again without reviving it." Now we do not find this to be the case to any thing like the same extent with other bad effects of mercury, and, so far as it goes, it rather militates against the notion that mercurial influence is at the bottom of the symptom. Be this as it may, we will present Dr. Colles' concluding exhortations with respect to the plan to be adopted when this or other complications have occurred:—

"Let us then bear in mind these facts; that each and all of these untoward effects of mercury are owing to the first impression of this medicine on the system; that on the subsidence of this state, the mercury may be used as freely and as long as can be required for the treatment of any venereal symptom, (or indeed for the cure of any disease curable by mercury,) without the danger of reproducing the same condition. Let us then, during the early period of a mercurial course, say from the second to the twelfth day, anxiously watch and guard against these untoward occurrences; but as soon as we have brought the system once fairly under the influence of this medicine, let us dismiss all fears and anxiety on this head, and now direct our whole attention to the changes in the venereal symptoms, to the degree of salivation, and to the strength and general health of our patients. The salivation once fairly established, we may consider ourselves as having escaped the chief dangers of a mercurial course, and as now being on the high road to a certain cure. We now may be confident that the mercury will not act (as it too often does) as a poison, instead of its proving one of the most active and beneficial remedies in the materia medica." 71.

We are as decided advocates for the use of mercury in syphilis as Dr. Colles is—as impressed as he, with the folly of prescribing an inadequate course. But we cannot go the length of reckoning salivation as the great safeguard against all accidents—no more than we could subscribe to the opinion that its presence is necessary for a cure. In reference to the latter point, we repeat that we continually see patients perfectly cured who cannot be fairly said to have exhibited any thing like ptyalism; in reference to the other point, we can positively affirm that some of the very worst cases we have seen of the bad effects of mercury have been those in which salivation *was* excited and maintained. Perhaps the ambiguity of language, and a certain difficulty in composition, may obscure or pervert the sentiments of

Dr. Colles, and may make him seem to say what he does not strictly mean. This is not improbable. But his less experienced readers will read the text as it is written, and attach to it no other sense than what appears upon the surface. We think it necessary to record our dissent from the apparent doctrine inculcated; because we believe that, if literally understood and unreservedly acted on, it would lead to unfortunate results.

There cannot, perhaps, be a more pregnant instance of what we must consider fanciful hypothesis, than the attempt of Dr. Colles to shew that the mercurial erethismus, occurs only in cases where ptyalism has not been obtained. We have lately witnessed a fatal instance of this very affection, now, fortunately rare. In that instance, a few doses of calomel excited a furious salivation, the mercurial erethismus followed, the pulse grew rapid and irregular, the limbs were affected with general tremors, the countenance was of a deadly white, the depression was excessive, and, in this condition, the patient died. Mr. John Pearson, who saw too many cases of the erethismus, for in his time mercury was given in large quantities, and the salivations were excessive, states that the subjects were men who had nearly, and sometimes entirely, completed their mercurial course. Is it likely that such men had not been affected, more or less, with ptyalism? On the whole, we conceive that our author's ideas on the sovereign virtues of ptyalism approach rather closely to what is called a crotchet.

This concludes the portion of the work devoted to the examination of the best mode of administering mercury for the cure of syphilis. The subject is important—too ill-understood, and too little considered by the bulk of medical men—beset with many difficulties at the best—obscured by prejudice, by ignorance, and by hypothesis—and in need of all the light that can be thrown on it by reasoning and by practical experience. An article on such a subject is not misplaced in the pages of a journal avowedly conducted on utilitarian principles. If inexperienced readers acquire any useful information, and if experienced ones are led to reflect more on what they see, and to communicate what they may learn, the purposes of utility will have been served.

